# The combined impact of chronic kidney disease and ulcer severity on incident cardiovascular events in patients with diabetes‐related foot ulceration

**DOI:** 10.14814/phy2.70415

**Published:** 2025-06-06

**Authors:** Nick S. R. Lan, Jonathan Hiew, Ivana Ferreira, J. Carsten Ritter, Laurens Manning, P. Gerry Fegan, Emma J. Hamilton, Girish Dwivedi

**Affiliations:** ^1^ Centre of Excellence for Cardiometabolic Health Fiona Stanley Hospital Perth Australia; ^2^ Department of Cardiology Fiona Stanley Hospital Perth Australia; ^3^ Medical School The University of Western Australia Perth Australia; ^4^ Harry Perkins Institute of Medical Research Perth Australia; ^5^ Centre of Excellence Multidisciplinary Diabetes Foot Ulcer Service Fiona Stanley and Fremantle Hospitals Group Perth Australia; ^6^ Department of Podiatry Fiona Stanley Hospital Perth Australia; ^7^ Medical School Curtin University Perth Australia; ^8^ Department of Vascular Surgery Fiona Stanley Hospital Perth Australia; ^9^ Department of Infectious Diseases Fiona Stanley Hospital Perth Australia; ^10^ Department of Endocrinology and Diabetes Fiona Stanley Hospital Perth Australia; ^11^ Victor Chang Cardiac Research Institute Sydney Australia

**Keywords:** cardiovascular diseases, chronic kidney disease, coronary artery disease, diabetes, foot ulcer, myocardial infarction

## Abstract

In patients with diabetes‐related foot ulceration (DFU), ulcer severity and chronic kidney disease (CKD) portend worse outcomes. We evaluated the combined impact of a severe ulcer with CKD on major adverse cardiovascular events (MACE) in patients with DFU. Ulcer severity was defined using the SINBAD (site, ischaemia, neuropathy, bacterial infection, area, depth) classification, dividing into low (1–3) or high (4–6). CKD was defined as an estimated glomerular filtration rate <60 mL/min/1.73 m^2^. Patients were categorized into four groups based on SINBAD and CKD category. MACE was defined as hospitalization for myocardial infarction, stroke or transient ischemic attack, or heart failure. Of 497 patients, 236 (47.5%) were SINBAD‐low/CKD‐absent, 80 (16.1%) SINBAD‐high/CKD‐absent, 127 (25.6%) SINBAD‐low/CKD‐present, and 54 (10.9%) SINBAD‐high/CKD‐present. The median follow‐up was 410 (interquartile range 242–576) days for MACE and 387 (221–549) days for MACE or all‐cause mortality. SINBAD‐high/CKD‐present was associated with significantly higher MACE (SINBAD‐low/CKD‐absent 4.2%; SINBAD‐high/CKD‐absent 7.5%; SINBAD‐low/CKD‐present 9.5%; SINBAD‐high/CKD‐present 33.3%; log‐rank *p* < 0.001) and MACE or all‐cause mortality (SINBAD‐low/CKD‐absent 5.5%; SINBAD‐high/CKD‐absent 13.8%; SINBAD‐low/CKD‐present 17.4%; SINBAD‐high/CKD‐present 53.7%; log‐rank *p* < 0.001). SINBAD‐high/CKD‐present was associated with MACE (*p* < 0.001) and MACE or all‐cause mortality (*p* < 0.001) after multivariate adjustment. Severe DFU with CKD amplifies the risk of MACE in patients with DFU, suggesting important implications for cardiovascular risk assessment.

## Graphical abstract

Low SINBAD was defined as a score of 1–3, high SINBAD was defined as a score of 4–6, and CKD was defined as an estimated glomerular filtration rate <60 mL/min/1.73 m^2^. CKD, chronic kidney disease; MACE, major adverse cardiovascular events; SINBAD, site, ischaemia, neuropathy, bacterial infection, area, depth. Created with BioRender.com

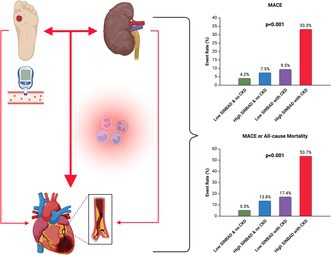

## 1 INTRODUCTION

Diabetes‐related foot ulceration (DFU) and chronic kidney disease (CKD) are serious complications of diabetes, each independently associated with a greater risk of cardiovascular disease (CVD), the leading cause of mortality among patients with diabetes. (Chin et al., 2024; de Boer et al., 2022; Gallagher et al., 2024; Jankowski et al., 2021; Valdivielso et al., 2019) Of over half a billion people living with diabetes worldwide, ~19%–34% will develop DFU and ~40% will develop CKD during their lifetime. (Armstrong et al., 2017; de Boer et al., 2022; GBD 2021 Diabetes Collaborators, 2023) Studies have demonstrated that in patients with DFU, CKD is a critical determinant of adverse outcomes such as delayed wound healing, lower extremity amputation, ulcer recurrence, cardiac events, and mortality. (Caruso et al., 2020; Ghanassia et al., 2008; He et al., 2017; Holman et al., 2024; Margolis et al., 2008; McDermott et al., 2023) Moreover, the severity of DFU is associated with adverse outcomes such as lower extremity amputation and mortality. (Brennan et al., 2017; Ha Van et al., 2023; Hamilton et al., 2021; Pickwell et al., 2015; Rubio et al., 2020) We have recently shown that in patients with DFU, those who have deep and/or infected ulcers are at a higher risk of major adverse cardiovascular events (MACE) compared with those who have ulcers that are not deep or infected (Lan, Hiew, et al., 2024).

Inflammation and infection in the context of DFU is hypothesized to hasten atherosclerosis and precipitate rupture of underlying plaque. (Lan, Hiew, et al., 2024; Libby et al., 2018; Prati et al., 2020) Patients with CKD can have more advanced atherosclerotic plaque and may thus be more susceptible to adverse sequelae from heightened inflammation. (Jankowski et al., 2021; Valdivielso et al., 2019) Notably, DFU and CKD share several risk factors and pathophysiological mechanisms, such as heightened systemic inflammation, oxidative stress, endothelial dysfunction, and a prothrombotic state, which may synergistically amplify the risk of CVD in patients with both conditions. (Jankowski et al., 2021; Lan, Dwivedi, et al., 2024) It is also possible that a bidirectional relationship exists between DFU and CKD, with each worsening the other. (Lan, Dwivedi, et al., 2024) Yet, the combined impact of both conditions on outcomes in patients with DFU has received limited attention. Given the potential implications on cardiac assessment and allocation of preventive therapies, we aimed to evaluate the combined impact of a severe ulcer with CKD on incident MACE in patients with DFU.

## 2 MATERIALS AND METHODS

This was a retrospective analysis of outpatients aged over 18 years who attended a multidisciplinary DFU service within a Western Australian tertiary hospital group between January 2022 and April 2024. Since January 2022, data for all new patients with DFU or existing patients with a new DFU are entered prospectively into a database for the Australian Diabetes Foot registry, as previously described. (Lan, Hiew, et al., 2024) Data pertaining to patient baseline characteristics, including demographics, comorbidities, medications, pathology results, and wound characteristics, were extracted from the local database. Additional data was obtained through review of electronic medical records, including hospital discharge summaries. Social deprivation index was determined using the Australian Socio‐Economic Indexes for Areas (SEIFA), where socioeconomic advantage and disadvantage are categorized into quintiles according to the patient's residential postcode. (Australian Bureau of Statistics, 2018) This research was approved by the South Metropolitan Health Service Human Research Ethics Committee with waiver of consent (RGS7053 and RGS4326).

The multidisciplinary DFU service is a National Association of Diabetes Centres (NADC) Centre of Excellence High Risk Foot Service and is staffed by endocrinologists, infectious diseases specialists, vascular surgeons, podiatrists, and an orthotist. The service routinely conducts a thorough wound assessment and classifies wounds using established classification systems as per guideline recommendations. (Hamilton et al., 2021) The wound assessment includes neurovascular assessment (peripheral pulses, toe pressures and sensation using monofilament and/or Ipswich touch test), measurement of dimensions (Silhouette Star camera; ARANZ Medical) and a depth assessment with probe‐to‐bone test. (Tan et al., 2024) For this study, the SINBAD (site, ischaemia, neuropathy, bacterial infection, area, and depth) wound classification system was utilized (see Table S1). (Hamilton et al., 2021) As a surrogate measure of wound severity, the SINBAD score was divided into a low score (a score between 1 and 3) or a high score (a score between 4 and 6), similar to a recently published study demonstrating that a high score significantly predicts major adverse foot events in patients with DFU. (Ha Van et al., 2023) In addition, our analysis of the receiver operating characteristic (ROC) curve with Youden's Index identified a SINBAD score of ≥4 as the optimal cut‐off for predicting MACE. For patients with multiple DFU, only characteristics pertaining to the most clinically important ulcer were considered for the analysis.

CKD was defined as an estimated glomerular filtration rate (eGFR) <60 mL/min/1.73 m^2^, as a persistent eGFR at this level is considered abnormal by guidelines (i.e., stage G3 CKD or greater). (de Boer et al., 2022) In addition, prior studies have demonstrated that an eGFR <60 mL/min/1.73 m^2^ is a predictor of adverse foot and/or cardiac outcomes in patients with DFU. (Ghanassia et al., 2008; He et al., 2017; Holman et al., 2024; Margolis et al., 2008) eGFR was calculated by our local laboratory from serum creatinine, age, and sex using the Chronic Kidney Disease Epidemiology Collaboration (CKD‐EPI) formula without race. (Levey et al., 2009) For this study, we further categorized the patients into four groups according to SINBAD score and eGFR categories: (1) low SINBAD score without CKD (SINBAD‐low/CKD‐absent); (2) high SINBAD score without CKD (SINBAD‐high/CKD‐absent); (3) low SINBAD score with CKD (SINBAD‐low/CKD‐present); and (4) high SINBAD score with CKD (SINBAD‐high/CKD‐present). Patients were excluded from the analysis if they had a diagnosis of Charcot foot but no DFU, a SINBAD score of 0, or no available eGFR result in the electronic medical records. The co‐primary outcomes of interest were as follows: (1) incident MACE only; or (2) a composite of incident MACE or all‐cause mortality. MACE was defined as the first occurrence of hospitalization for myocardial infarction, stroke, or transient ischaemic attack, or heart failure, including fatal and nonfatal occurrences, as previously described. (Lan, Hiew, et al., 2024) The status of the DFU at the time of the event or at the end of the follow‐up was also determined.

Statistical analyses were performed using SAS version 9.4 (SAS Institute Inc., USA) and SPSS version 29 (IBM, USA). Descriptive data are presented as mean ± standard deviation, count (percent), or median (quartile 1–3) where appropriate. Differences in baseline characteristics between the four groups were compared using analysis of variance (both parametric and non‐parametric where appropriate), Pearson's chi‐square test, or Fisher's exact test. ROC curves were constructed to determine whether models that included both the continuous SINBAD score and CKD stage better predicted the outcomes than either variable alone. Area under the curve (AUC) for ROC curves were compared using the method described by DeLong et al. (1988) Time‐to‐event data between the four groups were compared using the log‐rank test, and Kaplan–Meier curves were generated. Cox proportional hazards regression analyses were performed to estimate hazard ratios (HR) and 95% confidence intervals (CI) for the outcomes. Multivariate Cox regression analyses were performed adjusting for covariates with a *p* value <0.10 in baseline characteristics. These models were as follows: (1) unadjusted; (2) adjusted for age, type 1 diabetes, hypertension, ever‐smoking, sodium‐glucose cotransporter 2 (SGLT2) inhibitor use, glucagon‐like peptide‐1 (GLP1) agonist use, and glycated hemoglobin (HbA1c); and (3) adjusted for covariates in model 2 plus retinopathy, heart failure, any atherosclerotic CVD (coronary heart disease, peripheral arterial disease, or cerebrovascular disease), beta‐blocker use, and antiplatelet use. A two‐tailed *p* value <0.05 was defined as statistically significant.

## 3 RESULTS

Of 557 patients, 36 (6.5%) patients with a Charcot foot but no DFU, 8 (1.4%) with a SINBAD score of 0, and 16 (2.9%) with no available eGFR values were excluded. Of the 497 patients included, 134 (27.0%) patients had a high SINBAD score (4–6) and 181 (36.4%) were classified as CKD (eGFR <60 mL/min/1.73 m^2^). There was no significant difference in the proportion of patients with CKD in those with high versus low SINBAD score (*p* = 0.275). Overall, 236 (47.5%) patients were in the SINBAD‐low/CKD‐absent group, 80 (16.1%) were in the SINBAD‐high/CKD‐absent group, 127 (25.6%) were in the SINBAD‐low/CKD‐present group, and 54 (10.9%) were in the SINBAD‐high/CKD‐present group. Baseline characteristics according to SINBAD and CKD categories are summarized in Table 1. Significant differences between the four groups were observed for age (*p* < 0.001), hypertension (*p* = 0.006), ever‐smoking (*p* = 0.007), heart failure (*p* = 0.003), peripheral arterial disease (*p* < 0.001), prior percutaneous coronary intervention or coronary artery bypass graft surgery (*p* < 0.001), prior stroke or transient ischaemic attack (*p* = 0.014), retinopathy (*p* = 0.005), beta‐blocker use (*p* < 0.001), antiplatelet use (*p* < 0.001), and HbA1c (*p* < 0.001). C‐reactive protein level was significantly higher in patients with high SINBAD score (*p* < 0.001). There was a significant difference in ulcer status at follow‐up (*p* < 0.001), with a high SINBAD score being associated with lower rates of ulcer healing.

**TABLE 1:** Baseline characteristics according to SINBAD and eGFR categories^a^.

Characteristic	Low SINBAD and no CKD (*n* = 236)	High SINBAD and no CKD (*n* = 80)	Low SINBAD with CKD (*n* = 127)	High SINBAD with CKD (*n* = 54)	*p* Value
Age (years)	61.7 ± 12.3	64.1 ± 11.0	70.2 ± 12.1	69.2 ± 14.7	<0.001
Male sex	181 (76.7%)	65 (81.3%)	92 (72.4%)	38 (70.4%)	0.387
Indigenous	12 (5.1%)	7 (8.8%)	5 (3.9%)	2 (3.7%)	0.465
Lowest two deprivation quintile^b^	95 (40.6%)	39 (48.8%)	51 (40.5%)	24 (44.4%)	0.487
Type 1 diabetes	25 (10.6%)	15 (18.8%)	9 (7.1%)	4 (7.4%)	0.050
Hypertension	164 (69.5%)	48 (60.0%)	103 (81.1%)	42 (77.8%)	0.006
Dyslipidaemia	123 (52.2%)	47 (58.8%)	75 (59.1%)	36 (66.7%)	0.202
Ever‐smoker^b^	109 (71.7%)	46 (83.6%)	47 (60.3%)	22 (88.0%)	0.007
Heart failure	18 (7.6%)	7 (8.8%)	19 (15.0%)	13 (24.1%)	0.003
PAD	71 (30.1%)	48 (60.0%)	61 (48.0%)	48 (88.9%)	<0.001
Prior PCI/CABG	28 (11.9%)	15 (18.8%)	25 (19.7%)	20 (37.0%)	<0.001
Stroke/TIA	18 (7.6%)	12 (15.0%)	17 (13.4%)	12 (22.2%)	0.014
Peripheral neuropathy	218 (92.4%)	78 (97.5%)	120 (94.5%)	53 (98.1%)	0.248
Retinopathy	48 (20.3%)	22 (27.5%)	44 (34.6%)	21 (38.9%)	0.005
SGLT2 inhibitor	71 (30.1%)	18 (22.5%)	32 (25.2%)	7 (13.0%)	0.060
GLP1 agonist	63 (26.7%)	14 (17.5%)	31 (24.4%)	6 (11.1%)	0.054
Statin	155 (65.7%)	52 (65.0%)	92 (72.4%)	41 (75.9%)	0.307
ACEI/ARB	146 (61.9%)	43 (53.8%)	78 (61.4%)	33 (61.1%)	0.625
MRA	17 (7.2%)	4 (5.0%)	10 (7.9%)	7 (13.0%)	0.387
Beta‐blocker	70 (29.7%)	22 (27.5%)	62 (48.8%)	31 (57.4%)	<0.001
Antiplatelet	81 (34.3%)	42 (52.5%)	70 (55.1%)	36 (66.7%)	<0.001
Anticoagulant	36 (15.3%)	20 (25.0%)	24 (18.9%)	13 (24.1%)	0.175
eGFR	>90 (85–>90)	>90 (85–>90)	43 (28–50)	35 (17–46)	<0.001
CRP (mg/L)	10.5 (4.2–37.5)	57.5 (22.0–161.0)	14.0 (4.2–32.0)	93.5 (26.0–174.0)	<0.001
HbA1c (mmol/mol)	74.2 ± 23.7	78.1 ± 27.2	63.8 ± 21.5	73.3 ± 24.4	<0.001
HbA1c (%)	8.9 ± 2.2	9.3 ± 2.5	8.0 ± 2.0	8.9 ± 2.2	
LDL‐C (mmol/L)	2.0 ± 0.9	2.2 ± 1.1	1.9 ± 0.9	1.8 ± 1.1	0.136
Ulcer status at follow‐up^c^					
Not healed	52 (22.4%)	32 (40.0%)	35 (27.6%)	24 (44.4%)	<0.001
Healed	163 (70.3%)	32 (40.0%)	80 (63.0%)	18 (33.3%)	
Amputation	17 (7.3%)	16 (20.0%)	12 (9.4%)	12 (22.2%)	
Active ulcer at follow‐up^c^	26 (11.2%)	10 (12.5%)	16 (12.6%)	9 (16.7%)	0.749

*Note*: Data are presented as mean ± standard deviation, number (percent) or median (quartile 1–3).

Abbreviations: ACEI, angiotensin converting enzyme inhibitor; ARB, angiotensin receptor blocker; CABG, coronary artery bypass graft; CRP, C‐reactive protein; eGFR, estimated glomerular filtration rate; LDL‐C, low‐density lipoprotein cholesterol; MRA, mineralocorticoid receptor antagonist; PAD, peripheral arterial disease; PCI, percutaneous coronary intervention; SGLT2, sodium‐glucose cotransporter; TIA, transient ischaemic attack.

^a^ Low SINBAD was defined as a score of 1–3, high SINBAD was defined as a score of 4–6, and CKD was defined as an estimated glomerular filtration rate < 60 mL/min/1.73 m^2^.

^b^ Missing data for deprivation quintile for five patients and smoking in 187 patients.

^c^ At time of event or end of follow‐up; missing data for four patients.

**FIGURE 1:**
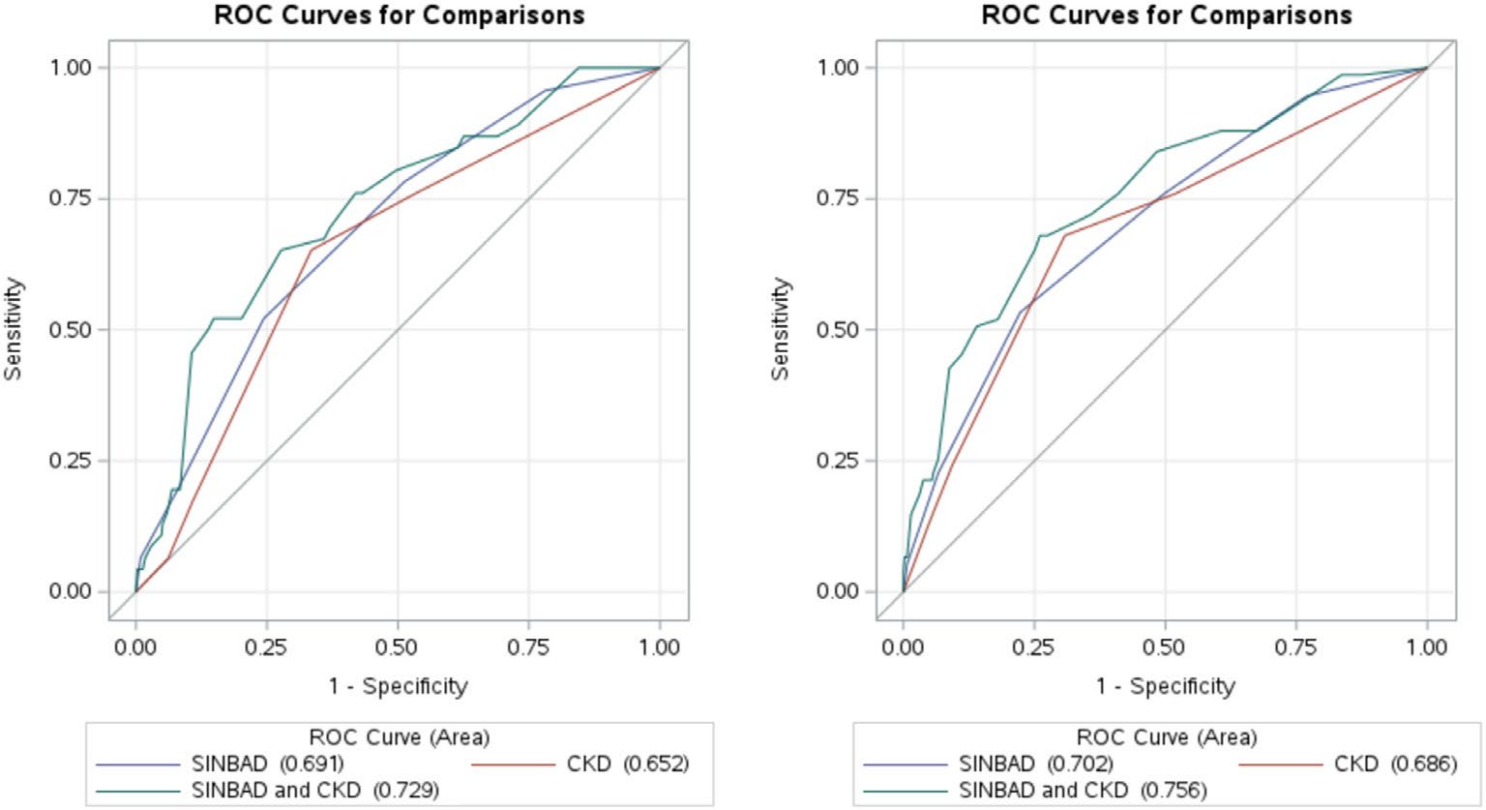
ROC curves for predicting MACE (left panel) and MACE or mortality (right panel) outcomes according to SINBAD score and CKD stage. ROC curves for models predicting MACE (left panel) and MACE or all‐cause mortality (right panel) for SINBAD score alone (continuous from 1 to 6), CKD stage alone (eGFR <15, 15–29, 30–59, 60–90, or ≥90 mL/min/1.73 m^2^) and both SINBAD score and CKD stage. All models significantly predicted the outcomes of interest (*p* < 0.05 for all). For MACE, the area under the ROC curve for the model that included both SINBAD score and CKD stage was significantly higher than that for SINBAD score alone (*p* = 0.047) and CKD stage alone (*p* = 0.001). For MACE or all‐cause mortality, the area under the ROC curve for the model that included both SINBAD score and CKD stage was significantly higher than that for SINBAD score alone (*p* = 0.004) and CKD stage alone (*p* = 0.017). CKD, chronic kidney disease; eGFR, estimated glomerular filtration rate; MACE, major adverse cardiovascular events; ROC, receiver operator characteristic; SINBAD, site, ischemia, neuropathy, bacterial infection, area, and depth.

The median follow‐up time was 410 (interquartile range 242–576) days for MACE only and 387 (221–549) days for MACE or all‐cause mortality. Incident MACE only occurred in 46 (9.3%) patients and incident MACE or all‐cause mortality occurred in 75 (15.1%) patients. MACE included 21 events (45.7%) of hospitalization for myocardial infarction (including three fatal cases), 20 events (43.5%) of hospitalization for heart failure, and five events (10.9%) of hospitalization for stroke or transient ischaemic attack (including one fatal stroke). MACE only and MACE or all‐cause mortality rates according to SINBAD score (1–6) or CKD stage (G1 to G5) are shown in Table S2a,b, respectively. SINBAD score (1–6) alone (*p* < 0.001) and CKD stage (G1–G5) alone (*p* = 0.001) significantly predicted MACE only, with AUCs of 0.691 and 0.652, respectively, as shown in Figure 1. The AUC for the model that included both SINBAD score and CKD stage in predicting MACE only was 0.729, which was significantly greater than the models for SINBAD score alone (*p* = 0.047) and CKD stage alone (*p* = 0.038). SINBAD score (1–6) alone and CKD stage (G1–G5) alone significantly predicted MACE or all‐cause mortality (*p* < 0.001 for both), with AUCs of 0.702 and 0.686, respectively. The AUC for the model that included both SINBAD score and CKD stage in predicting MACE or all‐cause mortality was 0.756, which was significantly greater than the models for SINBAD score alone (*p* = 0.004) and CKD stage alone (*p* = 0.017).

**FIGURE 2:**
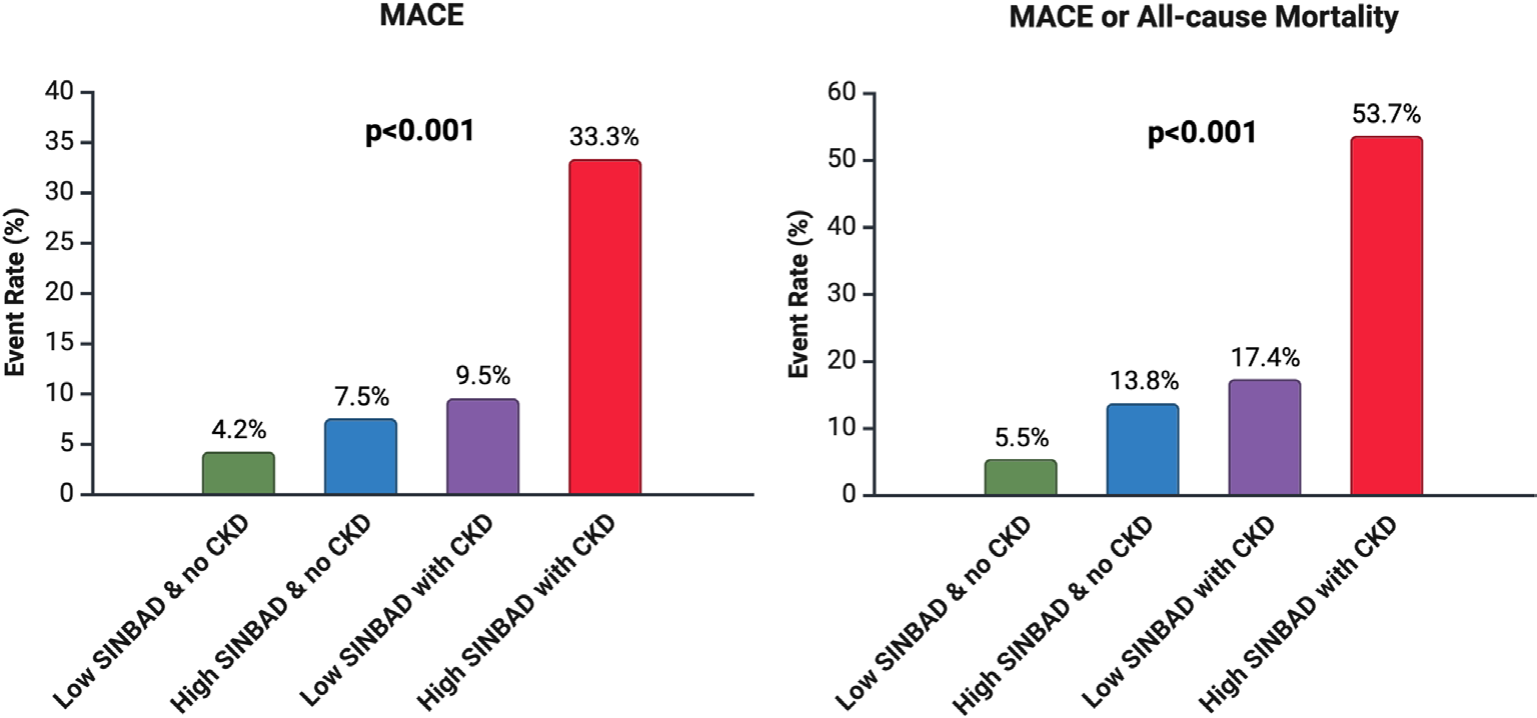
MACE and mortality outcomes according to SINBAD and CKD categories. Median follow‐up time 410 (242–576) days for MACE (left panel) and 387 (221–549) days for MACE or all‐cause mortality (right panel). Log‐rank *p* values show a significant difference in events between the four groups for both MACE and MACE or all‐cause mortality (*p* < 0.001 for both). Of 497 patients, there were 236 (47.5%) patients in the low SINBAD and no CKD group, 80 (16.1%) in the high SINBAD and no CKD group, 127 (25.6%) in the low SINBAD with CKD group, and 54 (10.9%) in the high SINBAD with CKD group. Low SINBAD was defined as a score of 1–3, high SINBAD was defined as a score of 4–6, and CKD was defined as an estimated glomerular filtration rate <60 mL/min/1.73 m^2^. CKD, chronic kidney disease; MACE, major adverse cardiovascular events; SINBAD, site, ischemia, neuropathy, bacterial infection, area, and depth. Created using BioRender.com.

The SINBAD‐high/CKD‐present group was associated with significantly higher rates of MACE only (SINBAD‐low/CKD‐absent 4.2%; SINBAD‐high/CKD‐absent 7.5%; SINBAD‐low/CKD‐present 9.5%; SINBAD‐high/CKD‐present 33.3%; log‐rank *p* < 0.001) and MACE or all‐cause mortality (SINBAD‐low/CKD‐absent 5.5%; SINBAD‐high/CKD‐absent 13.8%; SINBAD‐low/CKD‐present 17.4%; SINBAD‐high/CKD‐present 53.7%; log‐rank *p* < 0.001), as shown in Figure 2 and by the Kaplan–Meier curves (Figure S1). In Cox regression analyses, the SINBAD‐high/CKD‐present group was associated with a significantly higher rate of MACE only in the unadjusted model (*p* < 0.001) and adjusted models (*p* < 0.001 for models 2 and 3), as shown in Table 2. Similarly, the SINBAD‐high/CKD‐present group was associated with a significantly higher rate of MACE or all‐cause mortality in the unadjusted model (*p* < 0.001) and adjusted models (*p* < 0.001 for models 2 and 3). In the final adjusted model (model 3), the SINBAD‐high/CKD‐present group was associated with ~5 times the risk of MACE only (HR 5.15; 95% CI 2.13–12.43) and ~6 times the risk of MACE or all‐cause mortality (HR 6.39; 95% CI 3.12–13.11) compared with the SINBAD‐low/CKD‐absent group. Similar findings were observed when patients receiving dialysis (*n* = 27; 5.4%) were excluded from the analyses (see Tables S3 and S4).

**TABLE 2:** Multivariate Cox regression models for SINBAD and eGFR categories as a predictor of MACE and mortality outcomes.

Outcome	Group^a^	Model 1: HR and 95% CI	*p* Value	Model 2: Adjusted HR and 95% CI^b^	*p* Value	Model 3: Adjusted HR and 95% CI^c^	*p* Value
MACE	Low SINBAD and no CKD	Reference	<0.001	Reference	<0.001	Reference	<0.001
	High SINBAD and no CKD	1.69 (0.61–4.65)		1.75 (0.62–4.90)		1.40 (0.49–4.04)	
	Low SINBAD with CKD	2.20 (0.95–5.09)		1.47 (0.58–3.72)		1.13 (0.43–2.98)	
	High SINBAD with CKD	9.10 (4.20–19.73)		8.49 (3.67–19.65)		5.15 (2.13–12.43)	
MACE or all‐cause mortality	Low SINBAD and no CKD	Reference	<0.001	Reference	<0.001	Reference	<0.001
	High SINBAD and no CKD	2.47 (1.11–5.51)		2.08 (0.90–4.79)		1.64 (0.70–3.85)	
	Low SINBAD with CKD	3.21 (1.62–6.38)		2.27 (1.09–4.74)		1.80 (0.84–3.85)	
	High SINBAD with CKD	14.26 (7.39–27.52)		10.16 (5.10–20.26)		6.39 (3.12–13.11)	

Abbreviations: CI, confidence interval; eGFR, estimated glomerular filtration rate; HR, hazard ratio; MACE, major adverse cardiovascular event; SINBAD, site, ischaemia, neuropathy, bacterial infection, area, and depth.

^a^ Low SINBAD was defined as a score of 1–3, high SINBAD was defined as a score of 4–6, and CKD was defined as an estimated glomerular filtration rate <60 mL/min/1.73 m^2^.

^b^ Adjusted for age, type 1 diabetes, hypertension, ever‐smoker, SGLT2 inhibitor, GLP1 agonist, and HbA1c.

^c^ Adjusted for covariates in model 2 plus retinopathy, heart failure, any atherosclerotic cardiovascular disease (prior percutaneous coronary intervention or coronary artery bypass graft, peripheral arterial disease and stroke or transient ischaemic attack), beta‐blocker, and antiplatelet.

## 4 DISCUSSION

To the best of our knowledge, this is the first study demonstrating that in patients with DFU, the combination of a high SINBAD score with CKD is associated with an amplified risk of MACE, as well as MACE or mortality. While the SINBAD score and CKD alone were significant predictors of MACE, the combination of both demonstrated significantly greater predictive value. Moreover, after accounting for age and several confounding factors that are known to portend worse cardiovascular outcomes, there remained a ~5 times higher risk of MACE in patients with both a high SINBAD score and CKD compared with patients with a low SINBAD score and without CKD. The very high cardiovascular risk in patients with both a high SINBAD score and CKD is further evident by the observed MACE rate of ~30% and MACE or all‐cause mortality rate of ~50%, after a median follow‐up of just over 1 year. These alarming rates provide insight into this unique subgroup of patients with DFU, while suggesting a potential need for comprehensive cardiac assessment and the intensification of preventive therapies. Notably, prior studies in patients with DFU have demonstrated that overall mortality is ~14% and ~40% at 1 and 5 years, respectively. (Holman et al., 2024; Walsh et al., 2016) Our findings therefore suggest an urgent need for more comprehensive strategies that can mitigate morbidity and mortality due to CVD in this vulnerable cohort.

### 4.1 Pathophysiology

The development and progression of DFU, CKD, and CVD are interrelated, driven by shared risk factors (e.g., neuropathy) and pathophysiological mechanisms of systemic inflammation, vascular dysfunction, and metabolic derangements. (Jankowski et al., 2021; Lan, Dwivedi, et al., 2024; Tuttolomondo et al., 2015) The convergence of these pathways may potentiate each condition, while increasing the risk of future cardiovascular events. Indeed, worsening renal function is significantly associated with more severe diabetes‐related foot disease. (Bonnet et al., 2023; Wolf et al., 2009) Moreover, the incidence of acute kidney injury may be significantly higher in patients with DFU compared with patients with foot infection without diabetes, thereby contributing to the progression of CKD. (Ryan et al., 2022) CKD is characterized by a systemic and chronic pro‐inflammatory state that results in vascular and myocardial remodeling, thereby increasing the risk of cardiovascular events. (Jankowski et al., 2021; Valdivielso et al., 2019) The presence of DFU has also been shown to trigger an inflammatory cascade, characterized by marked upregulation of acute‐phase proteins, cytokines, and chemokines, with this being closely associated with ulcer severity. (Tuttolomondo et al., 2010; Weigelt et al., 2009) Indeed, our data demonstrated a higher C‐reactive protein level in patients with high SINBAD scores. Superimposed infection of DFU is common and might further trigger the immune system and exacerbate the pro‐inflammatory state. (Dietrich et al., 2017; Worsley et al., 2023) In aggregate, the coexistence of CKD and severe DFU may synergistically amplify cardiovascular risk, given that both conditions are independent predictors of cardiovascular events. Patients with CKD are likely to have more advanced atherosclerosis and may therefore be more vulnerable to plaque rupture from heightened systemic inflammation secondary to severe DFU. However, further studies and mediation analyses will be required to support these hypotheses.

### 4.2 Clinical implications

The poor outcomes faced by patients with both a high SINBAD score and CKD highlight several clinical implications. First, intensified management of modifiable risk factors such as dyslipidaemia, hypertension, obesity, glycemia, and lifestyle should be considered. Our data highlight that multimorbidity and other cardiovascular risk factors are prevalent in this cohort. Second, the therapeutic landscape for managing patients with diabetes and CKD is rapidly transforming and may be pertinent to this cohort. (Naaman & Bakris, 2023; Neuen et al., 2024, 2025) Along with angiotensin‐converting enzyme inhibitors or angiotensin II receptor blockers, SGLT2 inhibitors, GLP‐1 agonists, and finerenone have been shown to improve renal and cardiovascular outcomes in patients with type 2 diabetes and albuminuric CKD. (Bakris et al., 2020; Heerspink et al., 2020; Herrington et al., 2023; Perkovic et al., 2019, 2024; Pitt et al., 2021) Importantly, these therapies have mechanisms of action that target interrelated haemodynamic, metabolic, and inflammatory pathways that may be exacerbated in patients with DFU and CKD. (Naaman & Bakris, 2023; Ndumele et al., 2023; Neuen et al., 2024, 2025) Third, cardiac investigations may need to be considered in this high‐risk cohort to identify undiagnosed disease states that require treatment, thereby potentially reducing the risk of MACE. Fourth, strategies that can hasten wound healing, prevent infection, and lessen inflammation may need to be prioritized. (Golledge & Thanigaimani, 2021) In patients with end‐stage CKD, wound healing is impaired due to factors such as vascular calcification, anemia, uremia, nutritional deficiencies, oedema, neuropathy, and impaired immunity. (Bonnet & Sultan, 2022; Game et al., 2013) Lastly, the need to prevent DFU and CKD cannot be overstated.

### 4.3 Future research

Managing patients with DFU is complex, as the ulcer needs to be addressed alongside multiple comorbidities. Importantly, our findings concur with other studies demonstrating that CKD is a critical determinant of adverse outcomes in patients with DFU. (Caruso et al., 2020; Ghanassia et al., 2008; He et al., 2017; Holman et al., 2024; Margolis et al., 2008; McDermott et al., 2023) Hence, future clinical trials should evaluate the impact of intensive medical therapies for diabetic nephropathy and the role of anti‐inflammatory therapies in patients with DFU and CKD. The safety of SGLT2 inhibitors also requires clarification, given concerns about the increased risk of lower extremity amputations observed in the CANVAS trial and recent guidelines cautioning their use in patients with DFU. (Fitridge et al., 2024; Gallagher et al., 2024; Lan, Dwivedi, et al., 2024; Neal et al., 2017) Recent guidelines have also emphasized the pivotal role of kidney health in the prevention and management of cardiometabolic complications. (Ndumele et al., 2023) Future implementation research should be conducted to evaluate the impact of integrating novel cardiovascular‐kidney‐metabolic care models into multidisciplinary DFU services. (Lan, Dwivedi, et al., 2024) Importantly, a prior observational study has shown that the implementation of an intensive cardiovascular risk factor screening program and the use of risk‐reducing therapies was associated with a reduction in 5‐year mortality from 48% to 27% in patients with DFU (Young et al., 2008).

### 4.4 Limitations and strengths

Limitations of this study include the relatively modest sample size and the retrospective analysis of data from a single tertiary hospital group in Western Australia. Future studies with a multicenter prospective design with a longer a follow‐up duration will be required to confirm the findings of this study. Given the sample size and number of events, the results of the Multivariate Cox regression models should be interpreted with caution. The multivariate models did not account for several factors associated with CVD, such as physical activity, body weight, ethnicity, recreational drug use, other inflammatory conditions, and lipoprotein(a) levels, as data for these confounders were not available. Information on gradations of peripheral arterial disease severity were also not available. Furthermore, CKD was defined as an eGFR <60 mL/min/1.73 m^2^ on one measure, as data for repeated measures and albuminuria were not available. Cause of death could not be ascertained for many patients; this limited our ability to differentiate between mortality due to MACE versus other causes in outcome analyses and may have also resulted in an underestimated rate of MACE. Lastly, it should be noted that guidelines caution the use of classification systems such as SINBAD for predicting prognosis but recommend that individual components of the SINBAD classification system, rather than the total score, be used to facilitate communication between healthcare professionals. (Hamilton et al., 2021) However, the analysis of standardized data regarding wound characteristics and comorbidities that were prospectively collected in consecutive patients for a national registry strengthened the study. Furthermore, data was collected from the year 2022 onwards, therefore representing contemporary clinical practice and outcomes.

## 5 CONCLUSION

In patients with DFU, an already high‐risk cohort, those with the combination of a high SINBAD score and CKD represent a unique and susceptible group with a disproportionately higher risk of cardiovascular events. While both ulcer severity and CKD independently associate with adverse outcomes, their combined impact seems more substantial, suggesting an urgent need for targeted interventions, closer attention to underlying cardiac disease states, multidisciplinary management strategies, and upstream prevention. Further studies are needed to investigate whether targeted therapeutic approaches and the integration of models of care that address the complex interplay between DFU, CKD, and CVD can improve outcomes in this very high‐risk cohort.

## AUTHOR CONTRIBUTIONS

All authors were involved in the conception, design, and conduct of the study and interpretation of the results. N.S.R.L. was involved in the statistical analysis and wrote the first draft of the manuscript. All authors edited, reviewed, and approved the final version of the manuscript.

## FUNDING INFORMATION

Nick S. R. Lan is supported by a Western Australian Future Health Research and Innovation Fund, Athelstan Saw Clinician Researcher Training Scholarship, The University of Western Australia, and the South Metropolitan Health Service. This activity has been supported by the Western Australian Future Health Research and Innovation Fund (WA Near Miss Awards Emerging Leaders 2022, Emma J. Hamilton).

## CONFLICT OF INTEREST STATEMENT

NSRL has received research funding from Sanofi as part of a Clinical Fellowship in Endocrinology and Diabetes, education support from Amgen, AstraZeneca, Bayer, Boehringer Ingelheim, CSL Seqirus, Eli Lilly, Novartis, and Pfizer, speaker honoraria from Amgen, AstraZeneca, Boehringer Ingelheim, Eli Lilly, Menarini, Novartis, and Sanofi, and has participated in advisory boards for Eli Lilly. GD reports paid lectures from AstraZeneca, Pfizer, and Amgen and provides consultancy services, and has equity interest in Artrya Ltd.

## ETHICS STATEMENT

This research was approved by the South Metropolitan Health Service Human Research Ethics Committee with a waiver of consent (RGS7053 and RGS4326).

## Supporting information


Appendix S1.

## Data Availability

Data are available from the corresponding author upon reasonable request.
