# Supplementary material for: The combined impact of chronic kidney disease and ulcer severity on incident cardiovascular events in patients with diabetes‐related foot ulceration
Source: Physiol Rep. 2025 Jun 6;13(11):e70415. doi: 10.14814/phy2.70415 (PMC12141929; doi:10.14814/phy2.70415)
Supplement: Supplementary file 1 — Appendix S1. [file PHY2-13-e70415-s001.pdf]

**Supplemental Table 1 – SINBAD classification**

| **Category** | **Definition** | **Score** |
|---|---|---|
| Site | Forefoot<br>Midfoot and hindfoot | **0**<br>**1** |
| Ischaemia | Pedal blood flow intact: at least one palpable pulse<br>Clinical evidence of reduced pedal flow | **0**<br>**1** |
| Neuropathy | Protective sensation intact<br>Protective sensation lost | **0**<br>**1** |
| Bacterial infection | None<br>Present | **0**<br>**1** |
| Area | Ulcer $<1$ cm$^2$<br>Ulcer $\geq 1$ cm$^2$ | **0**<br>**1** |
| Depth | Ulcer confined to skin and subcutaneous tissue<br>Ulcer reaching muscle, tendon or deeper | **0**<br>**1** |
| Total possible score | | **6** |

Abbreviations: SINBAD site, ischaemia, neuropathy, bacterial infection, area, depth.

**Supplemental Table 2a – MACE and mortality outcomes according to SINBAD score**

| SINBAD | MACE rate | MACE or all-cause mortality rate |
|---|---|---|
| 1 (n=101) | 2 (2.0%) | 4 (4.0%) |
| 2 (n=129) | 8 (6.2%) | 14 (10.9%) |
| 3 (n=133) | 12 (9.0%) | 17 (12.8%) |
| 4 (n=89) | 15 (16.9%) | 23 (25.8%) |
| 5 (n=38) | 6 (15.8%) | 13 (34.2%) |
| 6 (n=7) | 3 (42.9%) | 4 (57.1%) |

**Supplemental Table 2b – MACE and mortality outcomes according to CKD stage**

| CKD stage | MACE rate | MACE or all-cause mortality rate |
|---|---|---|
| G1 (n=221) | 11 (5.0%) | 18 (8.1%) |
| G2 (n=95) | 5 (5.3%) | 6 (6.3%) |
| G3 (n=124) | 22 (17.7%) | 33 (26.6%) |
| G4 (n=26) | 5 (19.2%) | 8 (30.8%) |
| G5 (n=31) | 3 (9.7%) | 10 (32.3%) |

Estimated glomerular filtration rate (ml/min/1.73m$^2$) – G1 ≥90, G2 60-89, G3 30-59, G4 15-29, G5 <15.

**Supplemental Table 3 – MACE and mortality outcomes according to SINBAD and CKD categories following exclusion of patients receiving dialysis**

| **Outcome** | **Low SINBAD & no CKD (n=236)** | **High SINBAD & no CKD (n=80)** | **Low SINBAD with CKD (n=110)** | **High SINBAD with CKD (n=44)** | **Log-rank p-value** |
|---|---|---|---|---|---|
| MACE | 10 (4.2%) | 6 (7.5%) | 12 (10.9%) | 15 (34.1%) | <0.001 |
| MACE or all-cause mortality | 13 (5.5%) | 11 (13.8%) | 22 (20.0%) | 21 (47.7%) | <0.001 |

Abbreviations: CKD chronic kidney disease; MACE major adverse cardiovascular events; SINBAD site, ischaemia, neuropathy, bacterial infection, area, depth.

**Supplemental Table 4 – Multivariate cox regression models for SINBAD and eGFR categories as a predictor of MACE and mortality outcomes, where patients receiving dialysis were excluded from the analyses**

| Outcome | Group† | Model 1: HR and 95% CI | p-value | Model 2: Adjusted HR and 95% CI‡ | p-value | Model 3: Adjusted HR and 95% CI§ | p-value |
|---|---|---|---|---|---|---|---|
| **MACE** | **Low SINBAD & no CKD** | Reference | <0.001 | Reference | <0.001 | Reference | <0.001 |
| | **High SINBAD & no CKD** | 1.68 (0.61-4.63) | | 1.65 (0.59-4.61) | | 1.38 (0.48-3.95) | |
| | **Low SINBAD with CKD** | 2.55 (1.10-5.99) | | 1.64 (0.65-4.18) | | 1.35 (0.51-3.53) | |
| | **High SINBAD with CKD** | 9.91 (4.45-22.1) | | 7.92 (3.34-18.81) | | 5.29 (2.13-13.15) | |
| **MACE or all-cause mortality** | **Low SINBAD & no CKD** | Reference | <0.001 | Reference | <0.001 | Reference | <0.001 |
| | **High SINBAD & no CKD** | 2.47 (1.11-5.51) | | 2.02 (0.88-4.67) | | 1.58 (0.67-3.72) | |
| | **Low SINBAD with CKD** | 3.75 (1.89-7.46) | | 2.45 (1.17-5.13) | | 2.03 (0.95-4.36) | |
| | **High SINBAD with CKD** | 12.15 (6.07-24.32) | | 7.84 (3.78-16.28) | | 5.03 (2.37-10.68) | |

† Low SINBAD was defined as a score of 1-3, high SINBAD was defined as a score of 4-6 and CKD was defined as an estimated glomerular filtration rate <60 ml/min/1.73m$^2$.

‡ Adjusted for age, type 1 diabetes, hypertension, ever-smoker, SGLT2 inhibitor, GLP1 agonist and HbA1c.

§ Adjusted for covariates in model 2 plus retinopathy, heart failure, any atherosclerotic cardiovascular disease (prior percutaneous coronary intervention or coronary artery bypass graft, peripheral arterial disease and stroke or transient ischaemic attack), beta-blocker and anti-platelet.

Abbreviations: CI confidence interval; eGFR estimated glomerular filtration rate; HR hazard ratio; MACE major adverse cardiovascular event; SINBAD site, ischaemia, neuropathy, bacterial infection, area, depth.

**Supplemental Figure 1 – Kaplan-Meier curves for MACE and mortality outcomes according to SINBAD and CKD categories**

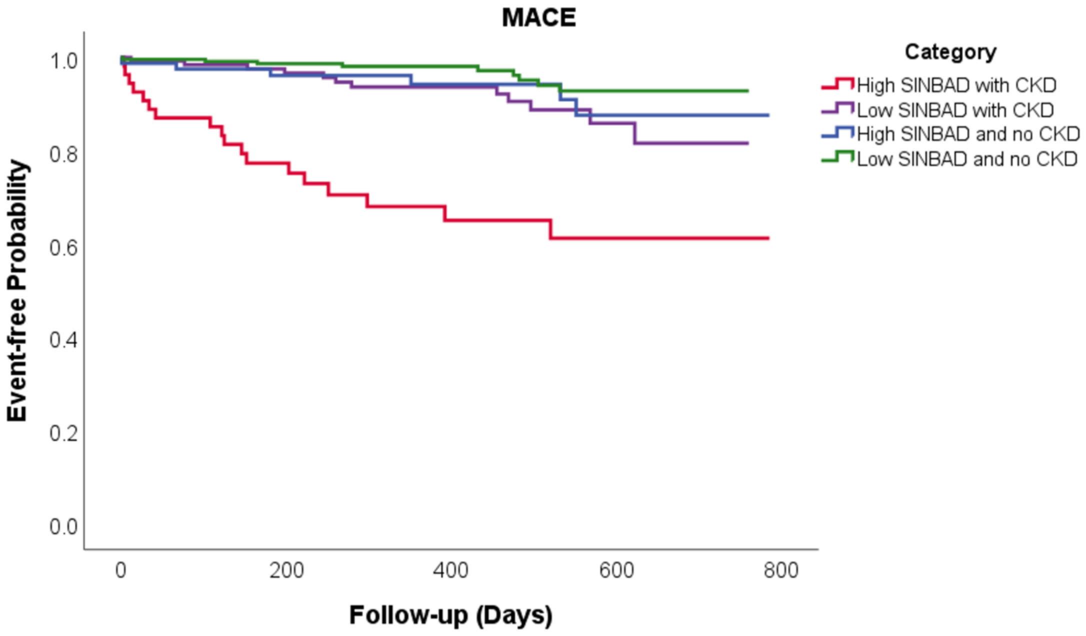


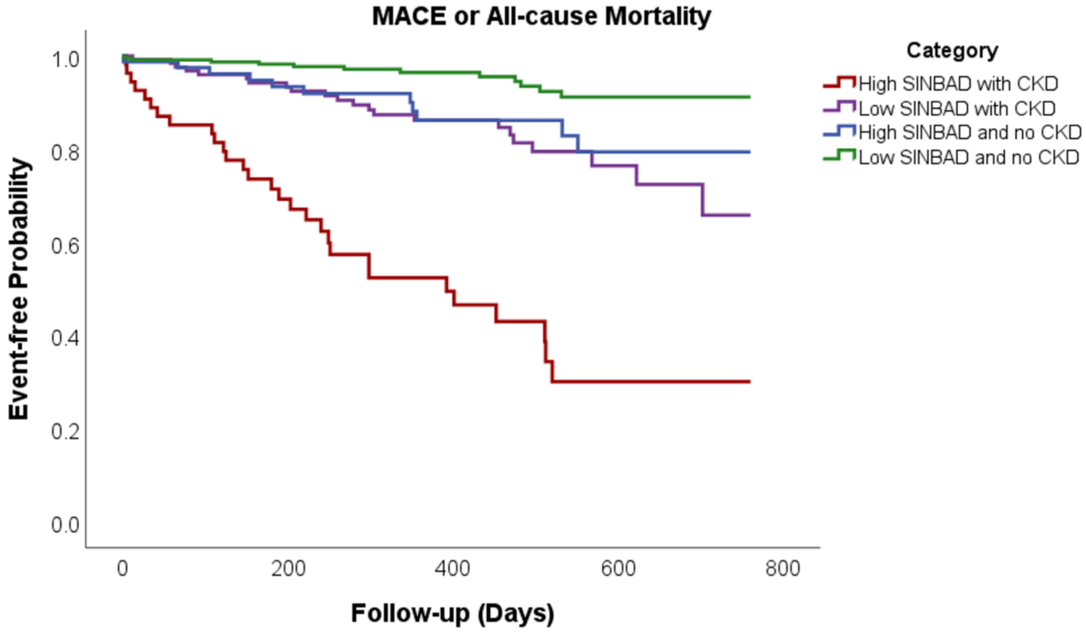


Figure legend – Median follow-up time 410 (242-576) days for MACE (top panel) and 387 (221-549) days for MACE or all-cause mortality (bottom panel). Log-rank p-value was <0.001 for both MACE and MACE or all-cause mortality, indicating a significant difference in events between the four groups. Low SINBAD was defined as a score of 1-3, high SINBAD was defined as a score of 4-6 and CKD was defined as an estimated glomerular filtration rate <60 ml/min/1.73m2.

Abbreviations: CKD chronic kidney disease; MACE major adverse cardiovascular events; SINBAD site, ischaemia, neuropathy, bacterial infection, area, depth.
